# Immunosenescence and the Geriatric Giants: Molecular Insights into Aging and Healthspan

**DOI:** 10.3390/medsci13030100

**Published:** 2025-07-28

**Authors:** Deasy Fetarayani, Mega Kahdina, Alief Waitupu, Laras Pratiwi, Mukti Citra Ningtyas, Galih Januar Adytia, Henry Sutanto

**Affiliations:** 1 Division of Allergy and Clinical Immunology, Department of Internal Medicine, Faculty of Medicine, Universitas Airlangga, Surabaya 60132, Indonesia; 2 Department of Internal Medicine, Dr. Soetomo General Academic Hospital, Surabaya 60286, Indonesia; drmegakahdina@gmail.com (M.K.); alief.waitupu-2022@fk.unair.ac.id (A.W.); laras.pratiwi-2022@fk.unair.ac.id (L.P.); mukti.citra.ningtyas-2022@fk.unair.ac.id (M.C.N.); galih.januar.adytia-2022@fk.unair.ac.id (G.J.A.); henry.sutanto-2022@fk.unair.ac.id (H.S.); 3 Internal Medicine Study Program, Department of Internal Medicine, Faculty of Medicine, Universitas Airlangga, Surabaya 60132, Indonesia

**Keywords:** aging, immunosenescence, SASP, geriatric giants, frailty, sarcopenia, inflammaging, immunology, geriatric medicine

## Abstract

Aging is associated with complex immune dysfunction that contributes to the onset and progression of the “geriatric giants”, including frailty, sarcopenia, cognitive decline, falls, and incontinence. Central to these conditions is immunosenescence, marked by thymic involution, the loss of naïve T cells, T-cell exhaustion, impaired B-cell class switch recombination, and increased autoreactivity. Concurrently, innate immunity deteriorates due to macrophage, neutrophil, and NK cell dysfunction, while chronic low-grade inflammation—or “inflammaging”—amplifies systemic decline. Key molecular pathways such as NF-κB, mTOR, and the NLRP3 inflammasome mediate immune aging, interacting with oxidative stress, mitochondrial dysfunction, and epigenetic modifications. These processes not only impair infection control and vaccine responsiveness but also promote tissue degeneration and multimorbidity. This review explores emerging interventions—ranging from senolytics and immunonutrition to microbiome-targeted therapies and exercise—that may restore immune homeostasis and extend healthspan. Despite advances, challenges remain in translating immunological insights into clinical strategies tailored to older adults. Standardization in microbiome trials and safety optimization in senolytic therapies are critical next steps. Integrating geroscience into clinical care could help to mitigate the burden of aging-related diseases by targeting fundamental drivers of immune dysfunction.

## 1. Introduction

Aging is accompanied by a set of complex clinical conditions known as the geriatric giants, which include frailty, falls, sarcopenia, cognitive impairment, immobility, and incontinence. The term “geriatric giants” was first coined by Bernard Isaacs (four Is: impairment of intellect [cerebral dysfunction], incontinence, immobility, and instability [falls]) to describe these conditions as major contributors to morbidity and functional decline in older adults [1,2]. These conditions often overlap, exacerbating each other and significantly increasing the burden on healthcare systems. Frailty, characterized by decreased physiological reserves, is strongly associated with sarcopenia—the progressive loss of muscle mass and strength—which itself contributes to falls and mobility impairment [3,4]. Cognitive decline and urinary incontinence further compound these issues, leading to reduced independence and quality of life [5,6]. One of the underlying drivers of these conditions is age-related immune dysfunction, commonly referred to as immunosenescence. Immunosenescence is characterized by diminished adaptive immunity, reduced T-cell diversity, and chronic low-grade inflammation, often termed inflammaging [7]. These immune alterations contribute to increased susceptibility to infections, impaired tissue repair, and systemic inflammation, all of which accelerate the progression of geriatric syndromes (Table 1). Additionally, oxidative stress, mitochondrial dysfunction, and the dysregulation of key molecular pathways, such as nuclear factor-kappa B (NF-κB) and mammalian target of rapamycin (mTOR), further exacerbate age-related immune decline [8,9,10]. Despite significant advances in our understanding of aging and immune function, there remain critical gaps in linking molecular and cellular immunology to clinical manifestations of the geriatric giants. This review aims to synthesize current knowledge on the immune mechanisms underlying these syndromes, explore their systemic implications, and highlight potential therapeutic approaches. By integrating insights from immunology and geriatrics, we seek to provide a comprehensive framework for future research and clinical interventions aimed at mitigating the impact of immunosenescence on aging populations.

## 2. Immunosenescence and Aging-Related Immune Dysregulation

### 2.1. Decline in Adaptive Immunity

#### 2.1.1. Thymic Involution

**Figure 1:**
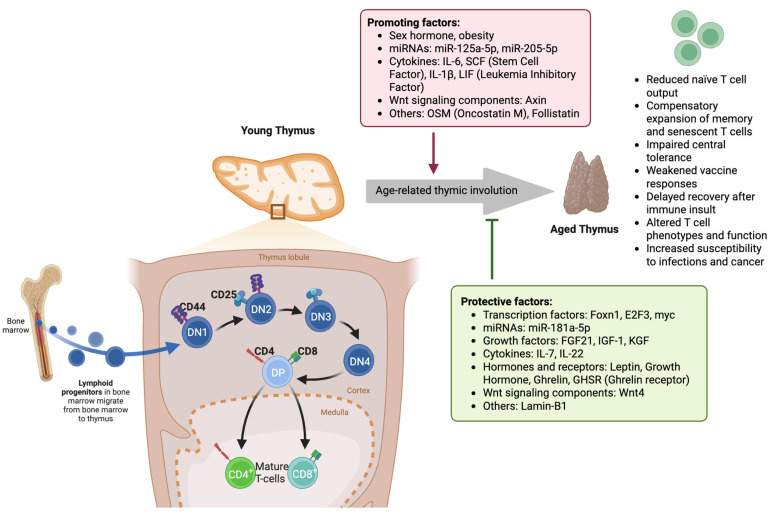
Molecular and cellular mechanisms underlying age-related thymic involution and its impact on T-cell development. In the young thymus, lymphoid progenitors from the bone marrow enter the thymic cortex and progress through distinct stages of thymocyte maturation: double-negative (DN1–DN4), double-positive (DP), and ultimately into mature CD4^+^ and CD8^+^ T cells in the medulla. Protective factors, such as transcription factors (Foxn1, E2F3, myc), miR-181a-5p, growth factors (FGF21, IGF-1, KGF), cytokines (IL-7, IL-22), and hormones like leptin, growth hormone, and ghrelin, along with Wnt4 signaling and Lamin-B1, maintain thymic structure and function. In contrast, age-related thymic involution is driven by promoting factors such as sex hormones, obesity, miR-125a-5p, miR-205-5p, inflammatory cytokines (IL-6, SCF, IL-1β, LIF), Wnt signaling component Axin, and other molecules, including OSM and Follistatin. Thymic involution results in reduced naïve T-cell output, the expansion of memory/senescent T cells, impaired central tolerance, weakened vaccine responses, delayed T-cell recovery post-insult, altered T-cell phenotypes, and increased susceptibility to infection and cancer. (DN: double-negative thymocyte (CD4^−^CD8^−^); DP: double-positive thymocyte (CD4^+^CD8^+^); CD: cluster of differentiation; FGF21: fibroblast growth factor 21; IGF-1: insulin-like growth factor 1; KGF: keratinocyte growth factor; IL: interleukin; SCF: stem cell factor; LIF: leukemia inhibitory factor; OSM: oncostatin M; Foxn1: forkhead box N1; E2F3: E2F transcription factor 3; myc: proto-oncogene c-Myc; GHSR: ghrelin receptor; Wnt: wingless/integrated signaling pathway).

Aging leads to profound changes in the adaptive immune system, primarily affecting T-cell function and diversity. One of the hallmark processes of immunosenescence is the decline in the production of naïve T cells due to thymic involution [11,12]. Thymic involution is driven by a combination of intrinsic and extrinsic factors that progressively deteriorate the thymic architecture and function (Table 2). The thymus, which is responsible for generating self-tolerant T cells, begins to shrink early in life, reaching a state of minimal functionality by middle age. This results in a sharp decline in thymopoiesis, leading to the reduced output of naïve T cells and the overrepresentation of memory and exhausted T-cell populations in the periphery (Figure 1) [13]. This process results in a decline in immune competence and increased susceptibility to infections, cancer, and autoimmune disorders. Thymic involution alters T-cell selection processes, leading to an increased risk of autoimmunity. As the negative selection of self-reactive T cells weakens with age, the potential for autoimmune reactivity increases, contributing to chronic inflammatory states associated with aging [14]. Several molecular pathways have been implicated in the regulation of age-related thymic atrophy, including transcription factor dysregulation, microenvironmental changes, cytokine imbalances, and metabolic shifts. The transcription factor forkhead box N1 (FOXN1) is a key regulator of thymic epithelial cell (TEC) maintenance and differentiation. Studies indicate that age-related decline in FOXN1 expression leads to the structural disorganization of the thymic microenvironment, resulting in impaired thymopoiesis [12]. A significant reduction in FOXN1 levels is observed during early thymic involution, leading to the loss of cortical and medullary TEC subsets that support T-cell maturation [15]. Additionally, reduced expression of autoimmune regulator (AIRE), another key transcription factor, contributes to the defective negative selection of self-reactive T cells, increasing the risk of autoimmunity in older adults [16,17].

Thymic involution is further exacerbated by the accumulation of senescent immune cells that secrete pro-inflammatory cytokines, amplifying inflammaging—a condition linked to many age-related diseases, including frailty, neurodegeneration, and cardiovascular disease [11]. Elevated levels of pro-inflammatory cytokines, including tumor necrosis factor-alpha (TNF-α), interleukin (IL)-6, and interferon-gamma (IFN-γ), contribute to TEC apoptosis and impaired thymocyte differentiation [22]. These cytokines activate the NF-κB signaling pathway, which enhances TEC senescence and accelerates thymic atrophy. Moreover, increased levels of transforming growth factor-beta (TGF-β) inhibit thymocyte proliferation and promote fibrosis within the thymic stroma [23]. Mitochondrial dysfunction and oxidative stress are other major contributors to thymic involution. The aging-associated accumulation of reactive oxygen species (ROS) damages TECs and disrupts thymic homeostasis. Increased oxidative stress impairs thymopoietic signaling pathways and promotes cellular senescence, leading to reduced thymocyte proliferation [24,25]. Additionally, age-related metabolic shifts result in lipid accumulation within the thymus, further impairing TEC function. The replacement of functional thymic tissue with adipose deposits disrupts T-cell development, reducing immune diversity [12]. Recent studies have identified several genetic factors that regulate thymic aging. The Fas signaling pathway, known for its role in apoptosis, is upregulated in aged TECs, leading to increased cell death and disruption of the thymic architecture [26]. Additionally, epigenetic modifications, including DNA methylation and histone modifications, alter the expression of genes involved in thymopoiesis. Age-related hypermethylation of FOXN1 has been linked to its reduced expression, further exacerbating thymic atrophy [27,28].

#### 2.1.2. Naïve T-Cell Reduction

Aging profoundly impacts the adaptive immune system, leading to a gradual decline in the number and function of naïve T cells, which are essential in responding to novel antigens. The reduction in naïve T cells in geriatric patients is driven by several molecular and cellular mechanisms, including the previously discussed thymic involution, homeostatic proliferation, transcriptional dysregulation, and metabolic shifts. Specifically, to compensate for the reduced thymic output, the homeostatic proliferation of existing naïve T cells occurs, driven by IL-7 and self-antigen stimulation [29]. However, this compensatory mechanism has unintended consequences. With repeated divisions, naïve T cells accumulate epigenetic modifications and telomere shortening, impairing their function [29,30,31]. Furthermore, homeostatic proliferation favors clonal expansion, reducing T-cell receptor (TCR) diversity and limiting the ability to recognize new antigens [32]. Aging also alters transcriptional programs that regulate naïve T-cell survival and function. Key transcription factors such as SMAD3 and BCL11A exhibit age-associated deregulation, impairing naïve T-cell quiescence and differentiation [33]. Additionally, epigenetic modifications, including the hypomethylation of pro-inflammatory genes and hypermethylation of IL-2 and TCR signaling genes, contribute to functional decline [34,35]. These changes reduce the ability of naïve T cells to respond effectively to new infections and vaccinations. Furthermore, metabolic reprogramming in aging naïve T cells contributes to their reduced survival and function. Aged T cells exhibit impaired glucose uptake, increased oxidative stress, and mitochondrial dysfunction, leading to apoptosis and senescence [36,37]. The accumulation of ROS damages cellular components, accelerating T-cell exhaustion and loss of immunocompetence [38].

#### 2.1.3. T-Cell Exhaustion

Aging is associated with progressive T-cell exhaustion, a dysfunctional state where T cells lose their ability to mount effective immune responses. T-cell exhaustion in geriatric patients increases the susceptibility to infections and diminishes vaccine efficacy. This process is driven by chronic antigen exposure, increased inhibitory receptor expression, metabolic dysfunction, and transcriptional reprogramming (Table 3). Aging is associated with persistent exposure to viral antigens (e.g., cytomegalovirus and Epstein–Barr virus) and tumor neoantigens, which drive chronic immune activation and the exhaustion of T cells. Continuous stimulation of the TCR leads to the progressive loss of effector functions, such as reduced cytokine production and proliferation capacity [39,40]. A key feature of exhausted T cells is the upregulation of inhibitory receptors, including programmed cell death protein 1 (PD-1), cytotoxic T-lymphocyte-associated antigen 4 (CTLA-4), lymphocyte activation gene-3 (LAG-3), and T-cell immunoglobulin and mucin-domain containing-3 (TIM-3) [41,42]. These receptors impair TCR signaling and T-cell activation, leading to functional exhaustion. PD-1, in particular, is a central regulator of exhaustion, inhibiting T-cell proliferation and cytokine release through SHP-2 phosphatase recruitment [43]. Moreover, exhausted T cells exhibit a distinct transcriptional and epigenetic landscape, which reinforces their dysfunctional state. The transcription factors T-bet and Eomesodermin (Eomes) play opposing roles in regulating exhaustion: while T-bet helps to maintain a pool of partially functional T cells, Eomes promotes terminal exhaustion [41]. Additionally, the transcription factor TOX (i.e., thymocyte selection-associated high mobility group box) is crucial for enforcing the exhausted phenotype by modifying chromatin accessibility [42]. Epigenetic modifications, such as DNA methylation and histone remodeling, permanently imprint the exhausted state. The promoters of genes encoding effector molecules (e.g., IFN-γ, IL-2, perforin) become hypermethylated, preventing their activation even after antigen clearance [44]. This explains why exhausted T cells fail to fully recover their function despite checkpoint blockade therapies.

Aging-related metabolic shifts also contribute significantly to T-cell exhaustion. Unlike functional T cells, which rely on aerobic glycolysis for rapid energy production, exhausted T cells exhibit mitochondrial dysfunction and defective oxidative phosphorylation (OXPHOS), leading to energy depletion [53]. Moreover, increased ROS production damages mitochondrial integrity and exacerbates T-cell dysfunction [54]. Exhausted T cells also exhibit impaired uptake of glucose and amino acids, further compromising their ability to sustain an immune response [55]. Inflammaging is also a hallmark of aging and exacerbates T-cell exhaustion. Elevated levels of pro-inflammatory cytokines (e.g., IL-6, TNF-α, and IFN-γ) promote persistent immune activation, driving T cells into a state of chronic stimulation and exhaustion [40]. In addition, regulatory T cells (Tregs) expand in aging, further suppressing T-cell function through IL-10 and TGF-β signaling [56]. This contributes to immunosenescence, reducing the immune system’s ability to mount effective responses against infections and tumors.

#### 2.1.4. Impaired Immunoglobulin Class Switch Recombination in Aging

B-cell aging plays a critical role in immunosenescence, contributing to increased susceptibility to infections, reduced vaccine efficacy, and heightened autoimmunity in older adults. A key feature of this decline is the impaired ability of B cells to undergo immunoglobulin (Ig) class switch recombination (CSR), a process necessary for generating high-affinity, isotype-switched antibodies such as IgG and IgA. This defect is primarily driven by the age-related downregulation of the E2A-encoded transcription factor E47 and activation-induced cytidine deaminase (AID), both of which are essential for CSR and somatic hypermutation (SHM). CSR is the process through which B cells switch from producing IgM antibodies to other isotypes (e.g., IgG, IgA, IgE), allowing antibodies to acquire specialized effector functions. This process depends on AID, which introduces double-strand DNA breaks at switch (S) regions in the immunoglobulin heavy chain (IgH) locus. These breaks are then repaired through non-homologous end joining, enabling the recombination of different constant-region genes with the variable region, thus changing the antibody isotype while preserving antigen specificity [57]. E47, a transcription factor encoded by the E2A gene, regulates AID expression. E47 binds to promoter regions of the *AICDA* gene (which encodes AID) and is necessary for initiating its transcription in activated B cells. In young individuals, stimulation through B-cell receptor (BCR), CD40, and cytokines like IL-4 upregulates E47, leading to robust AID production and effective CSR and SHM. However, in aged B cells, E47 expression is significantly reduced, resulting in less AID being produced [58]. This transcriptional downregulation is one of the main intrinsic defects that explains why elderly individuals produce fewer isotype-switched antibodies. The reduced expression of E47 and AID with age correlates strongly with lower levels of CSR, which can be measured by the reduced expression of circular transcripts (e.g., Igγ1 CTs) generated during recombination. Older adults show a decline in switched memory B cells (IgG^+^/IgA^+^/CD27^+^), a population generated through successful CSR and essential for rapid antibody responses upon re-exposure to pathogens or vaccination. Simultaneously, there is the accumulation of naïve B cells (IgG^−^/IgA^−^/CD27^−^), further diminishing the ability to generate high-affinity responses [59]. This impaired CSR also compromises SHM, the process by which point mutations are introduced into the variable region of Ig genes in germinal centers (GCs), refining antibody affinity through selection. Because AID is essential for both CSR and SHM, its reduced expression in aged B cells leads not only to isotype inflexibility but also to the production of low-affinity antibodies, impairing immune protection even when antibodies are present [60]. These changes directly impair the ability of the elderly to mount effective humoral responses, particularly against new or evolving pathogens.

#### 2.1.5. Generation of Autoreactive Antibodies in Aging

Another hallmark of B-cell aging is the increased generation of autoreactive antibodies. Under normal conditions, developing B cells in the bone marrow are subjected to central tolerance mechanisms that eliminate strongly self-reactive clones through receptor editing, deletion (apoptosis), or anergy (functional silencing). B cells that escape central tolerance migrate to peripheral lymphoid organs, where peripheral tolerance continues to enforce control via apoptosis, functional inactivation, or regulatory cell-mediated suppression. In aged individuals, both central and peripheral tolerance mechanisms become less effective. This inefficiency allows the increased survival of B cells with low to moderate self-reactivity. Studies have shown that age-related defects in apoptotic signaling, including impaired Fas/FasL pathways and reduced Bim expression, impair the deletion of autoreactive B cells. In autoimmune-prone models like Fas-deficient lupus mice (MRL-Faslpr), this failure allows self-reactive IgG-switched B cells to accumulate, particularly in GCs, exacerbating autoimmunity [61]. Second, GCs are specialized microenvironments in secondary lymphoid tissues, where B cells undergo SHM and CSR under the influence of T follicular helper (Tfh) cells. Normally, this process includes stringent selection to favor B cells that produce high-affinity antibodies to foreign antigens while removing those that gain autoreactivity due to mutations. With age, GCs become structurally disorganized and functionally compromised. This includes a reduction in the quality of Tfh cell help and an altered cytokine environment (e.g., more IL-10, less IL-21), which diminishes proper B-cell selection [62]. Consequently, autoreactive B cells are not efficiently culled during affinity maturation and can exit the GC as memory B cells or long-lived plasma cells. Third, class-switched memory B cells, particularly IgG^+^ cells, are more prone to autoreactivity than their unswitched IgM counterparts, partly because SHM introduces new specificities, including for self-antigens. Although class switching is essential for robust immunity, it also increases the risk of generating self-reactive clones, which normally would be eliminated. In younger individuals, IgA^+^ memory B cells are less autoreactive than IgG^+^ cells, possibly reflecting tighter control in mucosal compartments where IgA is generated. A study comparing IgG^+^ and IgA^+^ memory B cells in healthy humans found that IgA^+^ cells had significantly fewer autoreactive and polyreactive clones, suggesting that the isotype and environmental context influence self-reactivity control [63]. However, with aging, the impaired regulation in class-switched B-cell subsets, especially in IgG+ populations, results in the persistence of autoreactive memory cells, likely contributing to the increased levels of self-directed antibodies seen in older adults. Fourth, recent evidence suggests that autoreactive B cells exhibit distinct metabolic profiles that promote their survival. In lupus-prone mice, autoreactive B cells show increased glycolysis and oxidative phosphorylation compared to non-autoreactive counterparts. These metabolic adaptations help to sustain the energy demands of autoreactive cells during activation and antibody production. Inhibiting glycolysis with 2-deoxyglucose selectively reduced autoreactive B-cell responses in these models [64]. Thus, age-associated changes in cellular metabolism may enhance the survival and function of otherwise short-lived autoreactive B cells, reinforcing the autoimmune phenotype.

#### 2.1.6. Diminished Vaccine Responsiveness in Aging

Elderly individuals produce lower antibody titers following influenza vaccination, a phenomenon strongly associated with reduced AID expression and impaired CSR in activated B cells [60,62]. These defects are compounded by a reduction in B-cell diversity, increased proportions of dysfunctional “age-associated B cells”, and the skewing of antibody repertoires, which collectively reduce the specificity and durability of vaccine-induced protection [65]. In young individuals, the adaptive immune system maintains a broad and dynamic range of B-cell clones, generated through variable, diversity, and joining (V-D-J) recombination in the bone marrow. This diversity enables the recognition of a wide array of pathogens. However, in older adults, the bone marrow’s ability to produce new naïve B cells declines significantly. This decline is attributed to both intrinsic changes in hematopoietic stem cells and extrinsic shifts in the bone marrow microenvironment. As a result, the peripheral B-cell pool becomes dominated by memory and senescent clones that have limited flexibility. These long-lived clones occupy immunological niches and suppress the entry or expansion of new, potentially more effective B cells. The net effect is the contraction of the B-cell repertoire, which reduces the ability of the immune system to respond effectively to novel antigens, including those introduced by vaccination [66]. Compounding this issue is the expansion of a unique subset of dysfunctional cells termed age-associated B cells, or ABCs. These cells are characterized by altered surface markers (such as low CD21 and high CD11c), increased expression of the transcription factor T-bet, and a propensity to produce pro-inflammatory cytokines rather than participate in effective GC reactions. Unlike conventional memory B cells, ABCs do not efficiently differentiate into antibody-secreting cells upon stimulation. Instead, they accumulate in the spleen and peripheral blood in aged individuals and serve as poor precursors for high-affinity antibody responses. Their proliferation is often driven by chronic stimulation through toll-like receptors and inflammatory cytokines, linking their expansion to both aging and persistent infections. These cells represent a double burden: they occupy immunological space and contribute to systemic inflammation yet fail to deliver meaningful protective immunity [62]. In addition to reduced diversity and dysfunctional cell subsets, the antibody repertoire itself becomes skewed with age. Studies using high-throughput sequencing have shown altered usage of immunoglobulin heavy-chain variable (IGHV) gene segments, often favoring segments associated with lower-affinity or autoreactive antibodies [67]. There is also a shift in the isotype profile, with increased IgG2 and reduced IgG1 antibodies, which impacts functional properties such as complement activation and Fc receptor engagement. Moreover, the SHM process, which is essential in generating high-affinity antibodies during GC reactions, is compromised due to the reduced expression of AID. This leads to the production of antibodies that are both less specific and less effective in neutralizing pathogens. As a consequence, elderly individuals not only mount weaker responses to vaccines but also generate antibodies that decay more rapidly and provide shorter-lasting protection [65].

### 2.2. Alterations in Innate Immunity

Key innate immune components, including macrophages, neutrophils, and natural killer (NK) cells, exhibit significant age-related impairments, contributing to increased susceptibility to infections and chronic inflammation in older adults. These alterations are driven by changes in signal transduction, cytokine responses, metabolic dysregulation, and epigenetic modifications [68].

#### 2.2.1. Macrophage Dysfunction in Aging

Macrophage dysfunction in aging is driven by several interconnected factors, including epigenetic changes, mitochondrial dysfunction, metabolic shifts, and circadian dysregulation. Age-related alterations in microRNA (miRNA) expression, such as the decline of miR-146b, lead to dysregulated cytokine production and mitochondrial dysfunction, contributing to chronic inflammation [69]. Transcription factors like MYC and USF1 are downregulated, impairing phagocytosis, migration, and chemotaxis [70]. Mitochondrial dysfunction results in reduced energy production, increased oxidative stress, and excessive mitochondrial DNA (mtDNA) leakage, which activates the NLRP3 inflammasome and exacerbates inflammation [71]. Additionally, macrophages in aged individuals shift from OXPHOS to glycolysis, leading to inefficient energy use and increased pro-inflammatory cytokine production [72]. Dysregulated lipid metabolism results in ceramide and cholesterol accumulation, impairing inflammation resolution and contributing to cardiovascular disease [73]. Circadian rhythm disruptions, including reduced Kruppel-like factor 4 (KLF4) expression, weaken diurnal immune responses and impair cytokine regulation [74,75]. Furthermore, aging leads to a decline in autophagic flux, causing intracellular debris accumulation, increased inflammation, and impaired pathogen clearance [76]. Dysfunction of the p53 pathway further reduces apoptotic debris clearance, contributing to chronic inflammation and tissue degeneration [77].

The dysfunction of aged macrophages results in several detrimental effects on immune function and tissue homeostasis. Reduced phagocytic capacity and impaired antigen presentation weaken microbial recognition, increasing the susceptibility to infections [78]. Aged macrophages favor a pro-inflammatory (M1) phenotype, producing excessive IL-6, TNF-α, and IL-1β, which drive inflammaging and contribute to chronic inflammatory diseases [79,80]. Increased oxidative stress and ROS production impair cytokine signaling and antigen presentation, weakening adaptive immune responses [71]. Defective autophagy and impaired apoptotic cell clearance lead to the accumulation of cellular debris, triggering chronic inflammation and promoting autoimmune dysregulation [76]. The loss of circadian control over cytokine production results in inappropriate inflammatory responses, further increasing the risk of bacterial and viral infections [74]. Additionally, metabolic and lipid dysregulation in macrophages contribute to tissue damage and the progression of age-related diseases, including cardiovascular disorders [73]. These combined effects diminish macrophage-driven tissue repair and immune surveillance, accelerating aging-related immune decline and increasing the vulnerability to chronic diseases.

#### 2.2.2. Reduced Neutrophil Activity in Aging

Aging disrupts neutrophil function through metabolic dysfunction, impaired chemotaxis, defective phagocytosis, oxidative stress, and dysregulated apoptosis [81,82]. Metabolic alterations, including increased NAD+ levels and reduced ATP production, impair neutrophil activation and energy availability for immune responses [83]. A decline in glutathione levels weakens antioxidant defenses, making aged neutrophils more susceptible to oxidative stress, while decreased phospholipid metabolism compromises membrane integrity and receptor signaling, reducing responsiveness. Chemotaxis, a critical process for neutrophil migration to infection sites, is also altered with age. Upregulated CXCR4 expression promotes neutrophil retention in the bone marrow, while reduced L-selectin expression and impaired β2-integrin activation weaken adhesion and extravasation, delaying recruitment to inflamed tissues [84]. Additionally, aged neutrophils exhibit reduced Fc receptor (CD16) expression, impairing pathogen recognition and engulfment [85]. Decreased production of ROS, such as superoxide and hydrogen peroxide, reduces the bacterial killing capacity [86]. The diminished release of antimicrobial peptides, including defensins and cathelicidins, further weakens pathogen neutralization. Increased oxidative damage also contributes to neutrophil dysfunction, as hydrogen peroxide accumulation leads to cellular damage, while declining glutathione peroxidase activity heightens the susceptibility to oxidative stress [87]. Dysregulated apoptosis prolongs neutrophil survival, contributing to excessive inflammation and tissue damage [88].

The dysfunction of aged neutrophils results in increased susceptibility to bacterial and fungal infections, particularly pneumonia and urinary tract infections [89]. Impaired chemotaxis and migration reduce their ability to reach infection sites efficiently, leading to delayed pathogen clearance [90]. Reduced phagocytosis, oxidative burst, and antimicrobial peptide production prolong infection durations and weaken immune responses [91]. Dysregulated neutrophil apoptosis leads to excessive inflammation, contributing to chronic diseases such as atherosclerosis and neurodegeneration [92]. Additionally, prolonged inflammatory responses impair immune resolution, exacerbating tissue damage. Aging also weakens vaccine responses, reducing protection against infections like influenza and COVID-19 [89,93]. These combined effects compromise immune defense and contribute to age-related immune decline.

#### 2.2.3. Impaired NK Cell Function in Aging

NK cells are a critical component of the innate immune system, responsible for eliminating virus-infected and malignant cells. However, aging is associated with a decline in NK cell function, leading to increased susceptibility to infections, cancer, and autoimmune diseases [94]. Aging impairs NK cell function through alterations in cytokine signaling, subset redistribution, metabolic dysfunction, and inhibitory receptor upregulation. A significant reduction in IL-15 signaling, essential for NK cell survival, proliferation, and cytotoxicity, leads to decreased NK cell numbers, reduced granzyme B and perforin expression, and weakened IFN-γ production, impairing immune regulation [95,96]. NK cell population shifts occur with age, characterized by a decline in highly functional CD56^bright^ NK cells, an increase in mature CD56^dim^ NK cells, and the accumulation of terminally differentiated CD57+ NK cells, reducing immune adaptability and responsiveness [97,98,99]. Metabolic dysfunction further weakens NK cell activity, as increased ROS damage mitochondria, impairing energy production and viability [100,101]. Reduced glucose uptake and defective AMPK signaling suppress NK cell activation, leading to diminished degranulation and cytokine secretion [102]. Additionally, aging increases the expression of inhibitory receptors such as killer cell lectin-like receptor G1 (KLRG1), which suppresses NK cell activation by activating AMPK, reduces telomerase activity, and promotes NK cell senescence, further limiting immune responses [102].

The decline in NK cell function with aging results in reduced cytotoxicity, impaired cytokine production, and weakened immune surveillance [103]. Aged NK cells exhibit decreased perforin and granzyme production, reducing their ability to eliminate virus-infected and malignant cells [104]. The loss of CD56^bright^ NK cells and accumulation of CD56^dim^ NK cells weaken adaptive-like immune responses, further impairing tumor and infection control [105]. Additionally, reduced IFN-γ secretion compromises NK-mediated immune coordination, increasing the susceptibility to persistent viral infections such as herpesviruses and contributing to higher cancer incidences [106]. Weakened NK cell responses also reduce vaccine efficacy, limiting protection against infections in older adults.

### 2.3. Chronic Low-Grade Inflammation

**Figure 2:**
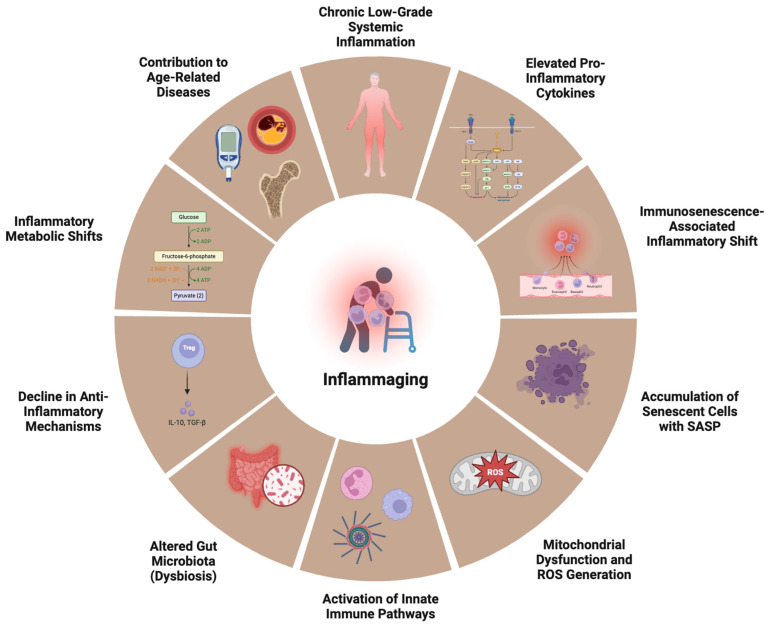
Hallmarks of inflammaging. Central to inflammaging is persistent chronic low-grade systemic inflammation, driven by elevated levels of pro-inflammatory cytokines and an immunosenescence-associated inflammatory shift—where aged immune cells acquire a pro-inflammatory phenotype. The accumulation of senescent cells secreting the senescence-associated secretory phenotype (SASP) further propagates inflammation. Mitochondrial dysfunction and the generation of reactive oxygen species (ROS) activate innate immune pathways, while aging-associated changes in the gut microbiota (dysbiosis) promote systemic exposure to microbial products like lipopolysaccharide (LPS). Simultaneously, there is a decline in anti-inflammatory mechanisms, such as the reduced secretion of IL-10 and TGF-β by regulatory T cells (Tregs). Inflammatory metabolic shifts, including increased glycolysis and altered lipid metabolism, support pro-inflammatory immune cell function. Collectively, these processes accelerate the onset and progression of age-related diseases, including cardiovascular disease, type 2 diabetes, osteoporosis, and neurodegeneration.

Aging is associated with a persistent, low-grade inflammatory state termed inflammaging, which contributes to age-related diseases such as cardiovascular disorders, neurodegeneration, metabolic syndrome, and cancer [107,108]. Unlike acute inflammation, which resolves after pathogen clearance, inflammaging is a chronic, sterile inflammatory response driven by persistent immune activation. This prolonged inflammatory state is fueled by senescent cell accumulation, dysregulated immune responses, and sustained exposure to endogenous and exogenous stressors [109]. The systemic effects of inflammaging are profound (Figure 2). Elevated levels of pro-inflammatory cytokines (IL-6, TNF-α, IL-1β, and IFN-γ) are observed in elderly individuals, leading to increased risks of atherosclerosis, type 2 diabetes, Alzheimer’s disease (AD), and osteoporosis [110]. Inflammaging also accelerates immunosenescence, weakening the immune response to infections and vaccinations [111].

The pathogenesis of inflammaging is driven by multiple molecular and cellular mechanisms, including oxidative stress, cellular senescence, epigenetic modifications, and the chronic activation of inflammatory signaling pathways [7,112,113]. Senescent cells accumulate with age and secrete a pro-inflammatory cocktail of cytokines, chemokines, and proteases, collectively known as the senescence-associated secretory phenotype (SASP). This includes IL-6, IL-8, CXCL10, and matrix metalloproteinases (MMPs), which perpetuate tissue inflammation and dysfunction [114]. Molecularly, SASP is regulated by several key signaling pathways, including NF-κB, p38 MAPK, and JAK/STAT, which respond to stress signals such as DNA damage, telomere shortening, and mitochondrial dysfunction. Activation of the cGAS-STING pathway, which detects cytoplasmic chromatin fragments, has also been identified as a key trigger of SASP, linking DNA damage to inflammatory responses [115]. Additionally, the NLRP3 inflammasome plays a role in amplifying SASP by inducing the release of IL-1β and IL-18, further sustaining inflammation [116]. In addition to SASP, aging-associated damage signals, such as damage-associated molecular patterns (DAMPs) and pathogen-associated molecular patterns (PAMPs), also activate the NF-κB signaling pathway, leading to the persistent transcription of pro-inflammatory genes [108]. Furthermore, NLRP3 inflammasome activation amplifies inflammaging by promoting IL-1β and IL-18 secretion, further driving chronic inflammation [117,118]. Meanwhile, mitochondrial dysfunction in aging cells leads to the excessive production of ROS, which damage DNA, proteins, and lipids, perpetuating inflammation [119]. This oxidative stress activates inflammatory cascades such as p38 MAPK, JNK, and NF-κB, further sustaining inflammaging [120].

## 3. Molecular Pathways in Age-Related Immune Dysfunction

### 3.1. Central Role of NF-κB, mTOR, and Inflammasomes in Immune Aging

Aging leads to profound immune dysregulation, with the chronic activation of pro-inflammatory pathways playing a significant role in immunosenescence. Three key molecular regulators—NF-κB, mTOR, and inflammasomes—orchestrate age-associated inflammation, oxidative stress, and immune dysfunction. Their dysregulation contributes to inflammaging, impaired adaptive immunity, and increased susceptibility to infections, cancer, and autoimmune disorders. First, NF-κB is a master regulator of inflammatory responses, controlling the transcription of cytokines, adhesion molecules, and survival factors. In aging, chronic NF-κB activation leads to sustained inflammation, contributing to immunosenescence and age-related diseases [121]. The constitutive activation of NF-κB in aging cells results from multiple stressors, including oxidative damage, mitochondrial dysfunction, and DNA damage. Additionally, declining SIRT1 and FOXO activity, which normally suppress NF-κB signaling, exacerbates inflammation [121,122]. miRNAs such as miR-146a further modulate NF-κB by targeting inflammatory gene transcripts, but their regulation declines with age [123]. The persistent activation of NF-κB disrupts immune homeostasis, driving the overproduction of IL-6, TNF-α, and IL-1β. This inflammatory cascade contributes to autoimmune disorders, cardiovascular disease, neurodegeneration, and frailty in the elderly [124]. Second, mTOR is a nutrient-sensing kinase that regulates cell growth, metabolism, and immune activation. With aging, the chronic activation of the mTORC1 complex skews immune responses, leading to increased inflammation and decreased immune regeneration [125]. Excessive mTOR activity in aged immune cells results in reduced autophagy, leading to the accumulation of damaged mitochondria and ROS production; increased pro-inflammatory cytokine secretion through NF-κB activation; and impaired T-cell differentiation, shifting the balance toward exhausted, dysfunctional T cells [126]. The pharmacological inhibition of mTOR using rapamycin has shown promise in reversing aspects of immune aging, enhancing autophagy, and improving vaccine responses in older individuals [127]. Third, inflammasomes, particularly NLRP3, are multiprotein complexes that mediate IL-1β and IL-18 production, fueling chronic inflammation. In aging, dysregulated NLRP3 activation amplifies immune dysfunction and contributes to metabolic and neurodegenerative diseases [128]. Aging-related inflammasome activation is driven by mitochondrial dysfunction and ROS production, which serve as danger signals; NF-κB-mediated priming, increasing pro-IL-1β expression; and loss of NAD+ and SIRT1 function, reducing the suppression of inflammasome activation [126]. Chronic NLRP3 activation contributes to tissue damage, neuroinflammation, and immune exhaustion, worsening frailty and disease progression in older adults.

### 3.2. Impacts of Oxidative Stress and Mitochondrial Dysfunction on Immune Cells

Aging is associated with progressive oxidative stress and mitochondrial dysfunction, both of which play central roles in immune system decline. Mitochondria are crucial for energy production and immune cell signaling, but their efficiency declines with age, leading to increased ROS production, impaired bioenergetics, and chronic inflammation. The accumulation of dysfunctional mitochondria contributes to immunosenescence, reducing the ability of immune cells to mount effective responses against infections, cancer, and chronic diseases [129]. One of the key consequences of mitochondrial dysfunction in aging immune cells is excessive ROS production, which leads to the oxidative damage of proteins, lipids, and DNA. In particular, mtDNA is highly susceptible to oxidative damage due to its proximity to the electron transport chain and lack of robust repair mechanisms. The accumulation of mtDNA mutations further impairs mitochondrial function, creating a vicious cycle of ROS production and cellular dysfunction [130,131]. In immune cells, this process disrupts T-cell activation, neutrophil chemotaxis, and macrophage phagocytosis, leading to a weakened immune response. T cells are particularly affected by mitochondrial dysfunction, as mitochondria regulate energy metabolism and calcium homeostasis, required for their activation and proliferation. Aged T cells exhibit reduced OXPHOS efficiency and ATP production, resulting in impaired proliferation and cytokine secretion. Furthermore, mitochondrial dysfunction skews T-cell differentiation toward pro-inflammatory and exhausted phenotypes, contributing to inflammaging and reduced adaptive immune responses [132,133].

Macrophages and dendritic cells are also highly sensitive to mitochondrial dysfunction. Aged macrophages exhibit reduced mitophagy—the process by which damaged mitochondria are cleared—leading to chronic inflammasome activation and the overproduction of IL-1β and IL-18. This enhances the NLRP3 inflammasome, a key driver of age-related inflammation [134,135,136]. Similarly, dendritic cells from older individuals have defective mitochondrial metabolism, impairing antigen presentation and reducing their ability to prime T cells against pathogens and tumors [137]. Therapeutic strategies targeting mitochondrial dysfunction and oxidative stress are being explored to rejuvenate immune function in aging. Approaches such as mitochondria-targeted antioxidants (e.g., MitoQ), caloric restriction, and exercise have shown promise in reducing oxidative stress, improving mitochondrial function, and enhancing immune responses [136,138].

### 3.3. Epigenetic Modifications Influencing Immune Responses

Epigenetic modifications play a crucial role in regulating immune function, affecting both innate and adaptive immunity. These modifications, which include DNA methylation, histone modifications, and non-coding RNA regulation, alter gene expression without changing the DNA sequence. With aging, epigenetic alterations contribute to immune dysfunction, inflammaging, and increased susceptibility to infections and chronic diseases [35]. DNA methylation, which typically represses gene expression, undergoes global hypomethylation with age, leading to genomic instability and increased inflammatory gene expression. At the same time, the hypermethylation of specific immune-related genes, including FOXN1 (essential for thymic function) and CD28 (critical for T-cell activation), results in reduced immune cell function [139,140]. These methylation changes impair T-cell proliferation, differentiation, and survival, weakening the adaptive immune response. In addition, the age-related hypomethylation of pro-inflammatory genes, such as those encoding IL-6, TNF-α, and IFN-γ, contributes to chronic inflammation and inflammaging [113,141].

Histone modifications, such as acetylation, methylation, and phosphorylation, influence the chromatin structure and gene expression. In aging, there is an imbalance in histone modifications that regulate immune genes, leading to immune dysfunction. Histone deacetylation at loci encoding cytokines reduces immune responsiveness, whereas histone acetylation at inflammation-related genes drives excessive cytokine production [142]. Furthermore, age-related changes in histone methylation impact T-cell differentiation. For example, the loss of H3K27me3, a repressive histone mark, results in the activation of inflammatory genes, while the gain of H3K4me3, an activating mark, enhances inflammatory signaling [143]. These alterations drive the pro-inflammatory polarization of macrophages and T cells, contributing to immune senescence. miRNAs and long non-coding RNAs (lncRNAs) also play essential roles in immune regulation. The age-related dysregulation of miRNAs, such as miR-146a and miR-155, impairs immune cell homeostasis. miR-146a, which normally suppresses NF-κB activation, declines with age, leading to uncontrolled inflammatory signaling and the hyperactivation of innate immune responses [144,145]. Conversely, the overexpression of miR-155 enhances pro-inflammatory cytokine production, exacerbating chronic inflammation. lncRNAs also modulate immune function by interacting with chromatin remodelers and transcription factors. Dysregulated lncRNA expression in aging has been linked to defects in T-cell activation, antigen presentation by dendritic cells, and macrophage polarization, further promoting immunosenescence [146,147]. Given that epigenetic modifications are reversible, epigenetic-targeted therapies hold promise in modulating immune aging. Histone deacetylase inhibitors (HDACis) and DNA methyltransferase inhibitors (DNMTis) are being explored to restore T-cell function and reduce inflammation [148]. Additionally, dietary interventions, such as polyphenol-rich diets, have been shown to modulate epigenetic marks and reduce age-related immune dysfunction [144,149].

## 4. Immune Contributions to Specific Geriatric Giants

### 4.1. Frailty and Sarcopenia

Frailty and sarcopenia are interconnected conditions in geriatric patients, characterized by the progressive loss of muscle mass, strength, and function, which increase their vulnerability to falls, disability, and mortality (Table 4). One of the major underlying mechanisms of sarcopenia is chronic inflammation, often driven by pro-inflammatory cytokines such as IL-6 and TNF-α [150]. These cytokines play a crucial role in promoting muscle catabolism, impairing muscle regeneration, and accelerating protein degradation, ultimately contributing to frailty and functional decline. IL-6, a key marker of inflammaging, is known to disrupt muscle homeostasis by promoting proteolysis via the ubiquitin–proteasome pathway and inhibiting insulin-like growth factor-1 (IGF-1) signaling, which is essential for muscle repair and growth [151,152,153]. Elevated IL-6 levels are associated with decreased handgrip strength, lower muscle mass, and increased fat infiltration, all of which characterize sarcopenia [154]. TNF-α is another potent driver of muscle wasting, acting through NF-κB signaling to upregulate the expression of atrogenes such as Muscle RING-Finger Protein-1 (MuRF1) and Atrogin-1, which mediate muscle protein breakdown [155]. TNF-α also induces mitochondrial dysfunction and oxidative stress, exacerbating muscle damage and reducing the regenerative capacity. Blocking TNF-α signaling with pharmacological inhibitors has been shown to prevent muscle atrophy and improve survival in aging models, highlighting its central role in sarcopenia pathogenesis [156]. Systemic inflammation is further amplified by senescent preadipocytes, which secrete high levels of IL-6 and TNF-α, exacerbating muscle degradation and impairing anabolic signaling [157]. Additionally, studies have identified C-reactive protein (CRP) and growth differentiation factor-15 (GDF-15) as key inflammatory markers that are elevated in frail individuals, further linking chronic inflammation to muscle loss [158].

### 4.2. Cognitive Impairment and Neuroinflammation

Neuroinflammation is a key driver of cognitive impairment in aging and neurodegenerative diseases such as AD. Central to this process are the dysregulation of microglial activation, the accumulation of amyloid-beta (Aβ) plaques, and disrupted immune signaling, which collectively contribute to neuronal damage, synaptic dysfunction, and cognitive decline (Table 4) [168]. Microglia, the resident immune cells of the brain, play a dual role in cognitive aging. In the early stages, microglia help to clear Aβ and support neuronal survival. However, in aging and neurodegenerative conditions, microglial overactivation leads to chronic inflammation, impairing their ability to clear Aβ and contributing to neuronal toxicity [169]. In AD, activated microglia cluster around Aβ plaques, releasing pro-inflammatory cytokines (e.g., IL-1β, TNF-α) and ROS, which exacerbate neuronal injury [161]. Amyloid-beta accumulation is a hallmark of neurodegeneration, and its interactions with immune cells worsen cognitive decline. Normally, microglia attempt to clear Aβ through phagocytosis, but, in aging, they lose their phagocytic capacity, leading to Aβ deposition and persistent inflammation [162,170]. Inflammatory pathways such as NF-κB and NLRP3 inflammasome activation further amplify Aβ toxicity by increasing cytokine production, promoting tau pathology, and accelerating neurodegeneration [171]. Neuroinflammation also disrupts synaptic function and neuronal communication, further impairing cognitive abilities. Studies using positron emission tomography (PET) imaging have shown that increased microglial activation correlates with cognitive decline and Aβ deposition in mild cognitive impairment (MCI) and AD [172]. Importantly, genetic studies have identified variants in microglial genes such as *TREM2*, which impair Aβ clearance and increase the AD risk [173,174]. Therapeutic interventions targeting neuroinflammation and microglial dysfunction are being explored to slow cognitive decline. Strategies such as enhancing microglial phagocytosis, inhibiting inflammatory pathways, and modulating gut microbiota-derived metabolites have shown promise in preclinical models [175,176].

### 4.3. Falls and Impaired Wound Healing

Aging is associated with a decline in immune surveillance and impaired tissue repair, which contribute to increased frailty, falls, and chronic wounds (Table 4). The immune system plays a central role in wound healing through the coordinated actions of neutrophils, macrophages, and cytokine signaling. However, in elderly individuals, these processes are dysregulated, leading to delayed wound closure, increased infection risks, and impaired tissue regeneration [177]. One of the primary defects in aging wound healing is the dysfunction of neutrophils and macrophages, which are critical for infection control and tissue remodeling. Aged neutrophils exhibit reduced chemotaxis, impaired phagocytosis, and delayed apoptosis, leading to prolonged inflammation and tissue damage [178]. Similarly, macrophages in elderly individuals fail to transition from a pro-inflammatory (M1) to a reparative (M2) phenotype, resulting in chronic inflammation, fibrosis, and impaired collagen deposition [179,180]. Additionally, the reduction in angiogenic growth factors such as vascular endothelial growth factor (VEGF) leads to poor neovascularization and delayed re-epithelialization. Aged skin exhibits decreased fibroblast proliferation and extracellular matrix remodeling, further impairing wound healing [181]. This results in a higher prevalence of chronic non-healing ulcers, particularly in frail individuals and those with comorbidities such as diabetes. Falls in older adults are also linked to weakened immune responses and chronic inflammation. The persistent activation of inflammatory pathways (e.g., NF-κB, NLRP3 inflammasome) not only impairs wound healing but also contributes to muscle weakness, joint instability, and frailty, increasing the fall risk [164,182].

### 4.4. Incontinence and Mucosal Immunity

Aging is associated with declining mucosal immunity in both the urinary and gastrointestinal (GI) tracts, leading to increased susceptibility to infections, incontinence, and chronic inflammation (Table 4). Changes in the urinary and gut microbiomes, along with the deterioration of epithelial barriers, contribute to dysfunction in maintaining homeostasis, making older individuals more prone to urinary tract infections (UTIs), gastrointestinal inflammation, and increased permeability of the gut and bladder walls [183]. The gut microbiota plays a central role in regulating immune function and maintaining intestinal epithelial integrity. With aging, the gut microbiota composition shifts toward increased pathogenic bacteria and reduced beneficial commensals, leading to gut barrier dysfunction. This phenomenon, often referred to as “leaky gut”, allows bacterial endotoxins such as lipopolysaccharide (LPS) to translocate into the systemic circulation, triggering inflammaging and immune dysregulation [184]. Age-related reductions in intestinal epithelial renewal and the decline in protective immune cells such as Th17 cells and secretory IgA further weaken mucosal defenses, increasing the susceptibility to gastrointestinal infections and chronic inflammation [165,185]. These changes in mucosal immunity may also contribute to incontinence by altering gut motility and disrupting neurological signaling between the gut and bladder. The urinary tract, previously thought to be sterile, hosts a unique microbiome that influences urinary health and immune responses. In older adults, the urinary microbiome undergoes significant shifts, characterized by a loss of *Lactobacillus* species and an increase in uropathogens such as *Escherichia coli*. This dysbiosis increases the risk of recurrent UTIs and bladder inflammation, which can exacerbate incontinence [186,187]. Bladder epithelial cells (BECs) serve as the first-line defense against bacterial invasion, but, in aging, BEC function declines, impairing their ability to expel pathogens. Uropathogenic bacteria can penetrate the weakened epithelial layer, forming intracellular reservoirs that evade immune clearance, leading to persistent infections and bladder dysfunction [166]. Aging also weakens tight junction proteins, such as occludin and claudin, in both the gut and bladder epithelia, increasing permeability and systemic inflammation [167,188]. This barrier dysfunction leads to the increased exposure of immune cells to microbial metabolites and endotoxins, triggering excessive cytokine production and further impairing tissue integrity. Additionally, chronic systemic inflammation exacerbates neuromuscular dysfunction, impairing bladder and bowel control, further contributing to incontinence in aging populations [189].

## 5. Potential Immunomodulatory Interventions

### 5.1. Anti-Inflammatory and Senolytic Therapies

Emerging therapeutic strategies, including anti-inflammatory drugs and senolytic therapies, aim to mitigate these effects by targeting pro-inflammatory pathways and selectively eliminating senescent cells. These approaches hold promise in restoring immune homeostasis, enhancing vaccine responses, and improving overall health in aging populations [190]. Anti-inflammatory drugs have been extensively studied for their roles in reducing systemic inflammation and improving immune resilience. Non-steroidal anti-inflammatory drugs (NSAIDs) such as aspirin and ibuprofen have been shown to reduce IL-6 and TNF-α levels, potentially delaying age-related immune decline. However, long-term NSAID use carries risks of gastrointestinal and cardiovascular complications [125,191]. mTOR inhibitors (e.g., rapamycin) have demonstrated the ability to suppress inflammaging while enhancing T-cell responses, making them promising candidates for boosting vaccine efficacy in older adults [192]. These therapies target key inflammatory pathways but may require long-term administration and precise dosing to maximize the benefits while minimizing side effects. Recently, chimeric antigen receptor (CAR) T-cell therapy has been explored to selectively target senescent immune cells, enhancing immune surveillance and reducing inflammaging [190,193].

Senolytics are a novel class of drugs designed to selectively eliminate senescent cells, which accumulate with age and secrete inflammatory cytokines, exacerbating immune dysfunction (Table 5) [194,195,196,197,198,199,200,201,202,203,204,205,206]. Senescent cells contribute to chronic inflammation through SASP, which includes IL-6, TNF-α, and MMPs [207]. The combination of dasatinib and quercetin (D + Q) has been shown to clear senescent cells, reduce inflammation, and improve metabolic function in aging individuals [194]. Navitoclax and venetoclax are Bcl-2 inhibitors that trigger apoptosis in senescent cells, attenuating age-related inflammation and tissue dysfunction [208,209]. Early clinical trials of senolytics have reported improvements in frailty markers, immune function, and overall healthspan, although challenges remain in optimizing dosing strategies and minimizing off-target effects [195]. An animal study demonstrated that short-term anti-senescence interventions could effectively mitigate long-term radiation-induced frailty and functional decline in a pre-clinical mouse model, mimicking the premature aging phenotype observed in cancer survivors. Early treatment with senolytics (Navitoclax or D + Q) or the senostatic drug metformin significantly reduced frailty and improved muscle, liver, and cognitive function for up to a year without repeated dosing. Notably, even delayed senolytic treatment after frailty onset yielded measurable benefits [210]. Another animal study provided evidence that intermittent oral treatment with the natural flavonoid senolytic fisetin could effectively reduce age-related frailty and improve skeletal muscle strength in old mice. Fisetin treatment had no impact on young mice but significantly improved grip strength and reduced frailty in aged animals—effects comparable to those achieved through the genetic clearance of p16^+^ senescent cells and pharmacological senolytic ABT-263. A transcriptomic analysis of skeletal muscle revealed that fisetin modulates gene expression linked to cellular senescence, including the downregulation of Cdkn1a and Ddit4 [211]. Another preclinical study showed that D + Q effectively attenuated age-related frailty in the SAMP10 mouse model of brain aging. Compared to normal-aging SAMR1 controls, SAMP10 mice exhibited a higher frailty index and impairments in motor and cognitive function. D + Q treatment significantly improved these parameters, alongside reducing senescent changes in the hippocampus. In vitro, D + Q also alleviated senescence in muscle and neuronal cells exposed to oxidative stress. These findings suggest that senolytic therapy with D + Q may offer protective effects against frailty and age-related functional decline [212]. Furthermore, muscle precursor cells (MPCs) in CKD mice exhibited strong markers of senescence, including elevated SA-β-gal activity, γ-H2AX, and cell cycle inhibitors p21 and p16^INK4a^, all of which correlated with reduced muscle mass and function. Treatment with the senolytic D + Q for 8 weeks significantly reduced these senescence markers, suppressed proinflammatory SASP cytokines, and improved muscle size, strength, and histology [213]. A single-arm pilot study demonstrates that intermittent treatment with the senolytic combination D + Q over 12 weeks is feasible and safe in older adults at risk for Alzheimer’s disease. While overall cognitive improvements measured by Montreal cognitive assessment (MoCA) scores were modest and not statistically significant, individuals with lower baseline cognition showed significant gains. Notably, reductions in the inflammatory SASP marker TNF-α correlated with improvements in cognitive scores, suggesting a potential mechanism of action for D + Q [214].

### 5.2. Immunonutrition and Microbiome-Targeted Interventions

Nutrition and microbiome modulation in immune aging are gaining increasing attention as potential strategies to enhance immune resilience and mitigate inflammaging. Dietary components, probiotics, and microbiome-targeted interventions can influence immune function by modulating the gut microbiota composition, systemic inflammation, and immune cell metabolism. These approaches aim to restore immune homeostasis, improve vaccine responses, and reduce the incidence of age-related diseases [215]. Immunonutrition refers to the use of specific dietary components to modulate immune responses and inflammation. Certain nutrients, such as omega-3 fatty acids, polyphenols, and micronutrients (vitamins D, E, and zinc), play critical roles in enhancing immune surveillance, reducing oxidative stress, and modulating inflammatory pathways [216]. Omega-3 fatty acids suppress pro-inflammatory cytokines such as IL-6 and TNF-α by inhibiting NF-κB activation and promoting resolvins, which are lipid mediators that resolve inflammation. Polyphenols (e.g., resveratrol, curcumin, quercetin) reduce oxidative stress and inflammation by modulating the Nrf2 and AMPK pathways, which regulate mitochondrial function and immune metabolism [217]. Dietary polyphenols and plant-derived compounds, such as quercetin and resveratrol, also exhibit anti-inflammatory effects by modulating NF-κB and inflammasome activation, potentially delaying immune aging [218]. Vitamin D enhances immune function by promoting regulatory T-cell development, reducing inflammation, and improving antimicrobial peptide production. Studies suggest that dietary interventions targeting these nutrients improve immune function, reduce chronic inflammation, and enhance vaccine efficacy in older adults [219,220].

With aging, microbial diversity declines, favoring a pro-inflammatory profile characterized by an increase in pathobionts (e.g., *Enterobacteriaceae*) and a decrease in beneficial commensals (e.g., *Bifidobacteria* and *Akkermansia*) [221]. Microbiome-targeted interventions, including probiotics, prebiotics, and fecal microbiota transplantation (FMT), are being explored to counteract dysbiosis and enhance immune resilience in aging populations [222]. Restoring microbial balance through diet and targeted supplementation has shown promise in improving immune function and reducing inflammaging. Supplementation with specific bacterial strains such as *Lactobacillus* and *Bifidobacterium* has been shown to reduce systemic inflammation, enhance gut barrier integrity, and improve immune responses in older adults [223]. Non-digestible fibers that promote the growth of beneficial gut bacteria, such as inulin and fructooligosaccharides (FOS), enhance short-chain fatty acid (SCFA) production and regulate immune homeostasis. Emerging studies suggest that FMT from young donors may restore microbial diversity, reduce inflammaging, and improve immune function in aging models [224,225]. These approaches highlight the potential of microbiome modulation in reversing immune dysfunction, improving metabolic health, and promoting longevity [226].

### 5.3. Exercise and Lifestyle Modifications

Regular physical exercise and lifestyle modifications have been identified as effective strategies in counteracting immunosenescence by improving immune cell function, reducing systemic inflammation, and enhancing overall healthspan [227]. Moderate, regular exercise has been shown to have profound effects on both innate and adaptive immunity. One of the key benefits of exercise is its ability to reduce the levels of pro-inflammatory cytokines such as IL-6 and TNF-α, thereby mitigating inflammaging, the persistent low-grade inflammation associated with aging [228]. Exercise also enhances the function of NK cells, which play a crucial role in antiviral defense and cancer surveillance. Studies have shown that regular aerobic and resistance-based training improves T-cell proliferation and function (reducing the accumulation of exhausted or senescent T cells), neutrophil phagocytic activity (enhancing pathogen clearance), and vaccine efficacy (improving antibody responses in older adults) [229]. Additionally, exercise helps to regulate the metabolic activity of immune cells, preventing immune cell dysfunction linked to aging [230]. Beyond exercise, other lifestyle factors, such as nutrition, stress management, and sleep hygiene, play critical roles in maintaining a robust immune system. Chronic psychological stress can impair immune resilience by suppressing T-cell function and increasing inflammatory cytokines, while regular physical activity has been shown to counteract these effects [230]. Sleep is another essential factor, as disruptions in circadian rhythms weaken adaptive immunity and increase the risk of infections [231]. Studies have found that regular exercise improves sleep quality, reducing the impact of sleep deprivation on immune function [232,233].

## 6. Current Challenges and Future Directions

Despite advances in the understanding of immunosenescence and inflammaging, several key questions remain unanswered. One major gap is the heterogeneity of immune aging—why some older adults maintain robust immune responses while others experience severe immune decline. Genetic, environmental, and lifestyle factors likely contribute to this variability, but their precise interactions remain unclear [234,235,236,237]. Another critical area is the immune response to vaccines and infections in the elderly. Aging reduces vaccine efficacy, yet mechanisms beyond T-cell exhaustion and antibody decline are poorly understood. Personalized vaccination strategies based on immune profiling could improve outcomes, but research on optimal adjuvants and delivery methods is still in its infancy [238]. Finally, age-related changes in the gut and urinary microbiomes and their influence on immune homeostasis are not fully characterized. The cross-talk between mucosal immunity, microbiota dysbiosis, and systemic inflammation presents a potential avenue for novel interventions [221]. Next, the growing aging population worldwide also presents a challenge for healthcare systems, requiring a shift from disease-focused treatment to preventive and regenerative strategies. Current healthcare models are largely designed to manage acute illnesses rather than chronic, multi-system aging-related conditions, leading to inefficiencies and escalating costs [239]. To address these challenges, healthcare systems must integrate immunosenescence assessments into routine clinical care to identify individuals at risk of immune decline; adopt personalized immunotherapy approaches for vaccines, cancer treatment, and chronic inflammation; and expand translational research funding to accelerate the development of immunomodulatory interventions for aging. Additionally, public health policies should emphasize lifestyle interventions, such as exercise, nutrition, and microbiome modulation, to enhance immune resilience and reduce healthcare burdens.

The transition from basic immunology research to clinical applications in geriatric populations also faces multiple barriers, including regulatory challenges, limited funding, and the insufficient representation of older adults in clinical trials [240]. Many therapies designed for younger individuals may not be effective or safe in aging immune systems, necessitating age-specific drug development and testing [241]. The emerging field of translational geroscience seeks to apply discoveries in aging biology to clinical interventions. Targeting fundamental aging mechanisms—such as cellular senescence, mitochondrial dysfunction, and immune dysregulation—may yield therapies that not only improve immune function but also extend healthspan and quality of life [242]. Personalized medicine approaches, including biomarker-based immunomodulation, are also gaining traction. By stratifying patients based on immune signatures, clinicians may be able to tailor interventions such as anti-inflammatory drugs, senolytics, or microbiome-targeted therapies for optimal efficacy. To clinically validate microbiome interventions, rigorous and reproducible methods are essential. These include identifying reliable biomarkers to stratify patients into responders and non-responders, which may depend on an individual’s baseline microbiota composition [243]. For example, patients with distinct microbial profiles might respond differently to probiotics, dietary fiber, or FMT, emphasizing the need for personalized approaches [244]. Clinical trials should integrate shotgun metagenomics and metabolomics to better characterize microbial functionality and its influence on human physiology beyond taxonomic shifts [245]. Furthermore, trials must control for diet, antibiotics, and environmental factors that significantly affect microbial diversity. Despite promising findings, the translation of microbiome-modulating interventions remains hindered by limited large-scale controlled trials and methodological heterogeneity [246], underscoring the urgent need for standardization and regulatory clarity [247]. For senolytic therapies, clinical validation involves confirming that the selective elimination of senescent cells results in meaningful health benefits without harming normal cells. Early-phase human trials with D + Q have demonstrated some functional improvements and biomarker shifts, particularly in idiopathic pulmonary fibrosis and Alzheimer’s disease [248,249]. Since lifespan endpoints are not feasible in human trials, alternative clinical trial designs are used to assess effects on frailty, multimorbidity, or resilience in aging-like conditions [250]. Promising new agents such as Seno-7284 have shown benefits in animal models of cardiometabolic disease and aging but remain to be validated in humans [204]. A critical aspect of clinical translation is avoiding off-target effects, especially given the essential role of senescence in tissue repair and tumor suppression [251]. By targeting aging-related multimorbidity, the trial could help to determine whether these therapies synergistically improve resilience and healthspan.

## 7. Conclusions

Aging profoundly impacts the immune system, driving a cascade of molecular and cellular dysfunction that contributes to the onset of the geriatric giants. Immunosenescence, chronic inflammation, and impaired immune resilience collectively influence frailty, cognitive decline, and other age-associated conditions, underscoring the need for targeted interventions. While recent advances have deepened our understanding of immune aging, many questions remain regarding the precise mechanisms and potential therapeutic strategies. Addressing these gaps through interdisciplinary research will be key to developing innovative approaches that enhance immune function and promote healthier aging. By bridging fundamental immunology with clinical applications, this field holds promise in improving outcomes and quality of life in aging populations.

## Figures and Tables

**Table 1:** Clinical problems included in geriatric syndrome.

Component of Geriatric Syndrome	Definition	Included in Geriatric Giants?
Frailty	A state of increased vulnerability due to a decline in physiological reserves and function across multiple organ systems, leading to increased risks of falls, hospitalization, and mortality.	Yes
Sarcopenia	Age-related loss of skeletal muscle mass, strength, and function, contributing to frailty, disability, and increased fall risk.	Yes (related to frailty)
Cognitive Impairment	Decline in cognitive function, including memory, executive function, and processing speed, often leading to dementia or mild cognitive impairment (MCI).	Yes
Falls	Unintentional loss of balance leading to collapse, often resulting from muscle weakness, poor coordination, and environmental hazards.	Yes
Immobility	Reduced ability to move independently due to musculoskeletal decline, neurological conditions, or chronic disease.	Yes (related to frailty and falls)
Incontinence	Loss of bladder and/or bowel control due to aging-related changes in the urinary system, pelvic floor dysfunction, or neurological conditions.	Yes
Delirium	Acute and fluctuating disturbance in attention and cognition, often triggered by infections, medications, or metabolic imbalances.	No
Depression	A common mental health condition in older adults, characterized by persistent sadness, loss of interest, and cognitive slowing.	No
Malnutrition	Deficiency in essential nutrients due to inadequate dietary intake, poor absorption, or chronic disease, leading to weight loss, muscle wasting, and increased morbidity.	No
Polypharmacy	The use of multiple medications (often ≥5), increasing the risk of drug interactions, adverse effects, and medication-related complications.	No
Osteoporosis	Age-related reduction in bone mass and density, increasing the risk of fractures and frailty.	No
Sleep Disorders	Includes insomnia, sleep apnea, and fragmented sleep patterns, affecting overall health and cognitive function in older adults.	No

**Table 2:** Key molecular mediators and cellular mechanisms implicated in thymic involution during immunosenescence.

Molecule/Factor	Role in Thymic Involution	Source/Target	Supporting Evidence
FOXN1	Downregulation of FOXN1 impairs thymic epithelial cell (TEC) maintenance, initiating thymic atrophy.	TECs	[12]
IL-7	Essential for T-cell development; reduced IL-7 availability leads to poor thymopoiesis.	TECs, thymocytes	[14]
IL-6, TNF-α, IL-1	Pro-inflammatory cytokines increase with age, contributing to thymic inflammation and involution.	Peripheral and thymic immune cells	[18]
IGF-1	Decreases with age; IGF-1 supports TEC survival and proliferation.	Systemic	[19]
miR-29a	Suppresses IFN-α receptor expression; protects thymus from infection-induced involution.	TECs	[20]
AIRE	Reduced expression impairs negative selection, allowing autoreactive T cells to escape.	mTECs	[18]
Sex Steroids (e.g., Estrogen, Androgens)	Promote thymic shrinkage; castration or blockade reverses involution.	Systemic	[11]
Reactive Oxygen Species (ROS)	Accumulation in TECs contributes to senescence and apoptosis.	TECs	[11]
Fatty Infiltration	Replaces thymic parenchyma, disrupting architecture and function.	Stromal replacement	[13]
Notch Pathway	Dysregulated Notch signaling affects thymocyte maturation.	TEC–thymocyte interaction	[21]
Bim (Pro-Apoptotic)	Downregulated in aged Tregs, leading to their accumulation and skewing of the immune balance.	Tregs	[18]

**Table 3:** Molecular mediators and pathways involved in T-cell exhaustion during immunosenescence.

Category	Molecule/Marker	Function/Role	Cell Type/Context	Supporting Evidence
Inhibitory Receptors	PD-1	Inhibits TCR signaling; hallmark of exhausted T cells during chronic stimulation	CD8^+^ T cells	[41]
	CTLA-4	Competes with CD28 for B7; suppresses early activation signals	CD4^+^ and CD8^+^ terminally differentiated effector memory T cells re-expressing CD45RA (TEMRA)	[45]
	KLRG1	Marker of terminal differentiation and reduced proliferative capacity	CD8^+^ T cells	[46]
	CD57	Linked to replicative senescence and cytotoxic potential; reduced proliferation	CD8^+^ memory T cells	[47]
	CD160	Suppresses TCR signaling; co-expressed in senescent-like cells	CD8^+^ clones in multiple myeloma	[48]
Transcription Factors	T-bet	Maintains a progenitor exhausted T-cell population; limits terminal exhaustion	CD8^+^ T cells	[41]
	Eomesodermin (Eomes)	Drives terminal differentiation of exhausted T cells	CD8^+^ TEX	[49]
	TOX	Regulates exhaustion-specific transcriptional programs	Exhausted CD8^+^ T cells	[46]
	NR4A1	Involved in establishing tolerance/exhaustion gene expression programs	CD8^+^ T cells	[46]
Phenotypic Markers	CD28^−^CD57^+^	Indicates senescent and exhausted phenotype with impaired proliferation	CD4^+^/CD8^+^ T cells	[50]
	CD27^−^CD28^−^	Terminally differentiated memory phenotype; prone to exhaustion	Effector memory T cells	[47]
Epigenetic Regulators	Exhaustion-specific chromatin	Stable epigenetic changes fix exhaustion phenotype, limiting reversibility	CD8^+^ exhausted T cells	[51]
Cytokine Environment	IL-6	Drives inflammaging; correlated with exhaustion and TEMRA skewing	Systemic/inflamed tissues	[45]
	Chronic antigen load (e.g., HIV)	Induces premature T-cell exhaustion and immunosenescence	All T-cell subsets	[52]

**Table 4:** Immune dysregulation in geriatric giants.

Geriatric Giant	Associated Immune Dysregulation	Mechanisms of Immune Dysfunction
Frailty [108]	Chronic low-grade inflammation (inflammaging)	↑ IL-6, TNF-α, and CRP promote systemic inflammation and catabolism
	T-cell exhaustion and immunosenescence	↓ Naïve T cells, ↑ CD8+ memory T cells, impaired adaptive immune response
	Mitochondrial dysfunction in immune cells	↑ Reactive oxygen species (ROS) production impairs immune metabolism and tissue repair
Sarcopenia [156,159,160]	Pro-inflammatory cytokines drive muscle catabolism	↑ IL-6, TNF-α, and IFN-γ induce muscle degradation via NF-κB
	Impaired macrophage function	↓ M2 (anti-inflammatory) macrophages lead to poor muscle regeneration
	Dysregulated autophagy	↓ Cellular clearance of damaged organelles impairs muscle repair
Cognitive Impairment [161,162,163]	Microglial overactivation and neuroinflammation	↑ IL-1β, TNF-α, and IL-6 contribute to synaptic dysfunction and neuronal loss
	Impaired amyloid-beta (Aβ) clearance	↓ Phagocytosis by aged microglia increases Aβ plaque accumulation
	Gut microbiome dysbiosis	↑ Pro-inflammatory gut microbes exacerbate neuroinflammation via the gut–brain axis
Falls [81,164]	Impaired neutrophil function	↓ Neutrophil chemotaxis and phagocytosis reduce healing capacity after falls
	Chronic inflammation and sarcopenia	↑ IL-6, TNF-α cause muscle weakness, increasing fall risk
Incontinence [165,166,167]	Decline in mucosal immunity	↓ Secretory IgA weakens bladder and gut epithelial defense
	Urinary microbiome dysbiosis	↓ *Lactobacillus* spp., ↑ uropathogens (*E. coli*) increase risk of UTIs
	Gut barrier dysfunction	↑ Intestinal permeability allows microbial translocation, leading to systemic inflammation

**Table 5:** Mechanisms of action, indications, and contraindications of senolytic drugs.

Senolytic Drug	Mechanism of Action	Indications	Contraindications
Dasatinib + Quercetin (D + Q)	Dasatinib inhibits BCR-ABL and Src family kinases, promoting senescent cell apoptosis. Quercetin is a flavonoid that inhibits PI3K, AKT, and anti-apoptotic pathways in senescent cells.	Idiopathic pulmonary fibrosis, age-related frailty, cardiovascular diseases, diabetes, osteoarthritis.	Thrombocytopenia, liver disease, bleeding disorders (dasatinib may cause platelet depletion). Avoid in patients on anticoagulants.
Fisetin	A flavonoid that inhibits mTOR and NF-κB signaling, promotes apoptosis of senescent cells, and reduces the senescence-associated secretory phenotype (SASP).	Neurodegenerative diseases (Alzheimer’s, Parkinson’s), cardiovascular aging, osteoarthritis.	Low bioavailability, high doses required for efficacy. Caution in kidney or liver dysfunction due to potential oxidative stress.
Navitoclax (ABT-263)	Bcl-2/Bcl-xL inhibitor that induces apoptosis of senescent cells by disrupting anti-apoptotic signaling.	Hematologic malignancies, fibrotic diseases, aging-related neurodegeneration.	Severe thrombocytopenia, gastrointestinal toxicity. Not recommended for use in patients with bleeding risks. Trabecular bone loss and impaired osteoprogenitor function in mice.
Seno-7284	Stimulates endogenous senolytic immune responses (NK and CD8+ T cells) via the Cxcl9–Cxcr3 axis, leading to the clearance of senescent cells.	Age-related cardiometabolic disorders, type 2 diabetes, atherosclerosis, progeroid syndromes.	Limited clinical data, potential immune overactivation. Avoid in patients with autoimmune diseases.
UBX-1325	Inhibits Bcl-xL, targeting senescent cells in diabetic retinopathy and age-related macular degeneration.	Diabetic macular edema, wet age-related macular degeneration.	Ongoing clinical trials, safety data limited. Avoid in patients with ocular infections.
Ginkgetin, Oleandrin, Periplocin	Machine learning-identified novel senolytics that disrupt anti-apoptotic pathways in senescent cells.	Aging-related metabolic disorders, cardiovascular disease.	Toxicity profiles not well established, need further validation before clinical use.

## Data Availability

No new data were created or analyzed in this study.
